# Significantly Elevated Levels of Plasma Nicotinamide, Pyridoxal, and Pyridoxamine Phosphate Levels in Obese Emirati Population: A Cross-Sectional Study

**DOI:** 10.3390/molecules25173932

**Published:** 2020-08-28

**Authors:** Ghada Rashad Ibrahim, Iltaf Shah, Salah Gariballa, Javed Yasin, James Barker, Syed Salman Ashraf

**Affiliations:** 1 Department of Chemistry, College of Science, UAE University, P.O. Box 15551, Al Ain, UAE; 201570102@uaeu.ac.ae (G.R.I.); altafshah@uaeu.ac.ae (I.S.); 2 Department of Internal Medicine, College of Medicine, UAE University, P.O. Box 15551, Al Ain, UAE; s.gariballa@uaeu.ac.ae (S.G.); javed.yasin@uaeu.ac.ae (J.Y.); 3 Department of Chemical and Pharmaceutical Sciences, Kingston University, Penrhyn Road, Kingston upon Thames, Surrey KT1 2EE, UK; j.barker@kingston.ac.uk; 4 Department of Chemistry, College of Arts and Sciences, Khalifa University, P.O. Box 127788, Abu Dhabi, UAE

**Keywords:** vitamins, obesity, Emirati population, bioanalytical quantification, serum

## Abstract

Water-soluble vitamins like B3 (nicotinamide), B6 (pyridoxine), and B9 (folic acid) are of utmost importance in human health and disease, as they are involved in numerous critical metabolic reactions. Not surprisingly, deficiencies of these vitamins have been linked to various disease states. Unfortunately, not much is known about the physiological levels of B6 vitamers and vitamin B3 in an ethnically isolated group (such as an Emirati population), as well as their relationship with obesity. The aim of the present study was to quantify various B6 vitamers, as well as B3, in the plasma of obese and healthy Emirati populations and to examine their correlation with obesity. A sensitive and robust HPLC-MS/MS-based method was developed for the simultaneous quantitation of five physiologically relevant forms of vitamin B6, namely pyridoxal, pyridoxine, pyridoxamine, pyridoxamine phosphate, and pyridoxal phosphate, as well as nicotinamide, in human plasma. This method was used to quantify the concentrations of these vitamers in the plasma of 57 healthy and 57 obese Emirati volunteers. Our analysis showed that the plasma concentrations of nicotinamide, pyridoxal, and pyridoxamine phosphate in the obese Emirati population were significantly higher than those in healthy volunteers (*p* < 0.0001, *p* = 0.0006, and *p* = 0.002, respectively). No significant differences were observed for the plasma concentrations of pyridoxine and pyridoxal phosphate. Furthermore, the concentrations of some of these vitamers in healthy Emirati volunteers were significantly different than those published in the literature for Western populations, such as American and European volunteers. This initial study underscores the need to quantify micronutrients in distinct ethnic groups, as well as people suffering from chronic metabolic disorders.

## 1. Introduction

It is well-established that vitamins and their metabolites are critical for cellular homeostasis, as well as cellular metabolism, mainly as coenzymes. For example, the phosphorylated forms of thiamine (vitamin B1) play a key role in the Krebs cycle [1,2], whereas riboflavin (vitamin B2), nicotinamide (vitamin B3), pantothenic acid (vitamin B5), pyridoxal 5-phosphate (circulating form of vitamin B6), 5-methyl tetrahydrofolate (circulating form of vitamin B9), and biotin (vitamin B8) are involved in oxidation/reduction reactions, fatty acid and neurotransmitter synthesis and metabolism [3,4,5,6]. Because all these vitamers are directly involved in various important cellular functions, their deficiency has a direct impact on human health. Supplementation is therefore highly recommended for targeted populations such as pregnant women, lactating women, infants, the elderly, and athletes to prevent various diseases, such as cardiovascular risk [7], anemia, cognitive impairment [8], and neural tube defects in newborns [9,10]. Vitamin B6 has also been shown to be important for normal cognitive function and in lowering the incidence of coronary heart disease among the elderly [11,12,13]. Supplementation also improves the physical performance of the same targeted population [14]. For example, vitamin B6 supplementation has been shown to reduce diabetic complications and incidences of neurodegenerative diseases in varying degrees [15]. The vitamin B6 group includes pyridoxal, pyridoxine, pyridoxamine, and their metabolites. The phosphate ester derivative pyridoxal 5′-phosphate (PLP) is the biologically active form of this vitamin [16] and reflects long-term body storage [17]. Studies have shown that low plasma PLP concentrations are associated with an increased risk of cardiovascular disease (CVD) [18,19]. Recent data have shown that plasma PLP is adversely associated with inflammatory markers, which include C-reactive protein, fibrinogen, and blood cell count [19,20,21,22] Additionally, low vitamin B6 concentrations are commonly present in diseases with a strong inflammatory basis, such as diabetes [23], rheumatoid arthritis [24], and inflammatory bowel disease [25]. Current evidence highlights the notion that inflammation may represent another link between vitamin B6 and CVD. However, the relationship of vitamin B6 status with inflammation and other CVD risk factors has not yet been extensively investigated in a population at high risk of CVD [26].

Niacin, also known as vitamin B3, is the precursor of the redox mediator coenzymes nicotinamide adenine dinucleotide (NAD) and nicotinamide adenine dinucleotide phosphate (NADP) [27]. Niacin has also been shown to decrease low-density lipoprotein cholesterol (LDL), very low-density lipoprotein cholesterol (VLDL-C), and triglycerides (TG), as well as to increase high-density lipoprotein cholesterol (HDL) levels [28]. Niacin alone [29] or in combination with other lipid-lowering agents such as statin [30] or ezetimibe [31] has been shown to significantly reduce the risk of cardiovascular disease and atherosclerosis progression [32]. There is some evidence that niacin might also help in lowering the risk of Alzheimer’s disease, cataracts, osteoarthritis, and type 1 diabetes [33]. Though the role of vitamin B6 in reducing complications associated with diabetes, aging, and neurodegenerative diseases has been widely reported [11,12,34,35], most of the published work on vitamin B6 is limited to general clinical observations and case studies.

In the past ten years, LC-MS/MS has been demonstrated to be uniquely suitable for the analysis of many water-soluble vitamins. However, only a few methods have been published for the multi-analyte quantification of water-soluble vitamins in complex biological matrices such as human milk [36,37], human plasma [38], and urine [39]. For instance, in 2012, Hampel et al. quantified four different water-soluble vitamins represented by five analytes in human milk by LC-MS/MS [40]. Thus far, an analytical approach for performing the simultaneous quantification of vitamins B1, B2, B3, B5, B6, B8, B9, and their main circulating forms in human plasma has not been published. This can be of great advantage when vitamer profiling is required in large longitudinal studies [41]. The objectives of the present work were two-fold: (1) to develop a sensitive robust and easy LC-MS/MS-based assay for measuring vitamins B6 and B3 in human plasma and (2) to use this method to measure plasma vitamins B6 and B3 in healthy and obese Emirati populations.

## 2. Results and Discussion

The most widely used variation of the LC-MS method are the tandem mass-spectrometry and “multiple reaction monitoring” (MRM) methods. This approach basically uses tandem mass-spectrometers to detect a specific product ion that is generated from a precursor ion (the parent compound) under a given set of fragmentation conditions. The monitoring of precursor-to-product ion allows for the specific and accurate determination of given analytes, even if they are not chromatographically resolved in the liquid chromatography part of the LC-MS/MS method. Initial experiments were conducted with pure analytical standards to identify the precursor and product ions for specific analytes. The vitamers, their structures, abbreviations, the mass-to-charge ratio (*m*/*z*) for the precursor and products ions, and the fragmentor voltage and collision energies values determined for the various vitamers are summarized in Table 1.

These parameters were then used to develop a short, 10-min, LC-MS/MS-based chromatographic method (described more under Materials and Methods) that was able to quantify the five different B6 vitamers, nicotinamide, and an internal standard (pyridoxine hydrochloride (PN)-D3).

**Figure 1:**
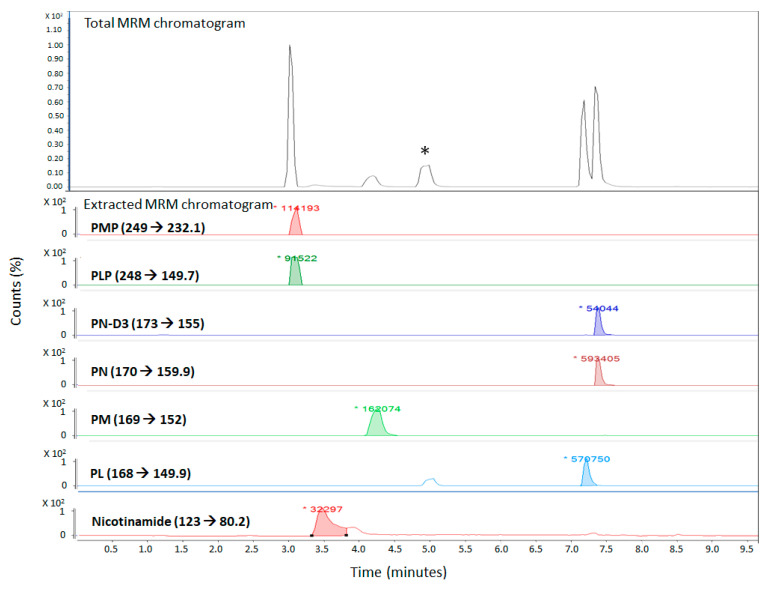
LC-MS/MS chromatogram of all analytes in the simulated plasma solution in the optimized LC-MS/MS method. (* refers to a peak which most likely is an isomer of PL as it is only seen in the extracted MRM chromatogram of PL (MRM 168→149.9).).

Since the final application of our assay was the analysis of vitamins B6 and B3 in human plasma, we also wanted to determine the best method for the extraction of these vitamins from plasma. The choice of precipitating agent [42,43] and incubation conditions [44] are known to greatly influence the effectiveness of protein precipitation and analyte extraction. TCA has been reported to be effective in precipitating proteins in human plasma [42,43]. However, it is also known that some vitamers, such as PLP, are strongly bound to plasma proteins [45,46] and may require vigorous vortexing as well as incubation conditions at 50 °C for their complete release. Therefore, experiments were conducted to determine which temperature incubation of the TCA-mixed samples would result in the highest recovery of our analytes from spiked simulated plasma. Three different conditions were chosen to be tested with 5 and 60 min incubations: 0 °C, room temperature, and 50 °C. Our results showed that 50 °C incubation of the TCA-precipitated samples for 5 min gave similar results to those incubated at room temperature or ice for one hour (Supplementary information, Table S1). Therefore, all the experiments shown here were performed using a 0.3 N TCA precipitation of each plasma sample at 50 °C for 5 min, followed by 1 min of vortexing and then centrifugation at 15,000× *g* for 10 min to obtain the supernatant that was then used for LC-MS/MS analysis, as shown in Figure 1. Table 2 shows the typical recovery of spiked vitamers obtained using our optimized TCA-based extraction in simulated plasma. Several mixtures of vitamers of different concentrations were then analyzed using the optimized LC-MS/MS method to establish standard curves for the five B6 vitamers as well as nicotinamide (B3). Table 3 shows the lower limit of detection (LLOD) and the lower limit of quantification (LLOQ) determined for each of the vitamers of interest under our optimized conditions. Further statistics of the partially validated method including precision, accuracy, and linear range are shown in Supplementary Table S2.

This optimized, robust, and relatively simple method was then used to screen plasma samples from 57 healthy and 57 obese Emirati volunteers. Table 4 shows the concentrations of the vitamers that were detected in the human plasma samples of healthy patients. Surprisingly, we were not able to detect pyridoxamine (PM) in any of the samples, while the other five vitamers were detected with varying concentrations. Table 4 (and Supplementary Figure S1a) also shows that the average concentration of the pyridoxamine-5′-phosphate (PMP) analyte in all 57 samples was around 30 nM, and the patient samples showed an even distribution around this average. The PLP analyte was only detected in the plasma samples of 14 patients, with an average concentration of 36 nM (Supplementary Figure S1b). On the other hand, the PN and pyridoxal hydrochloride (PL) analytes were detected in the plasma samples of all 57 patients, with average concentrations of 21 and 45 nM, respectively, (as shown in Figure S1c,d). Finally, the nicotinamide analyte was only detected in 54 samples, with an average concentration of 850 nM.

Similarly, a total of 57 human plasma samples of obese patients were also analyzed for the concentrations of the analytes in these samples. Table 5 (and Supplementary Figure S2) shows the analysis results of five analytes (PMP, PLP, PN, PL, and nicotinamide), while PM (just like in the healthy population) was not detected in any of the obese plasma samples. All plasma samples showed variable concentrations of PMP, with an average value of 50 nM, as shown in Figure S2a. On the other hand, the concentration of PLP was only detected in the plasma samples of eight patients with an average value of 37 nM (Figure S2b). The concentrations of PN and PL were detected in the plasma samples of all obese patients, with average concentrations of 21 and 61 nM, respectively (Figure S2c,d). Finally, the concentration of nicotinamide was detected in a total of 45 samples, with an average value of 3700 nM.

**Figure 2:**
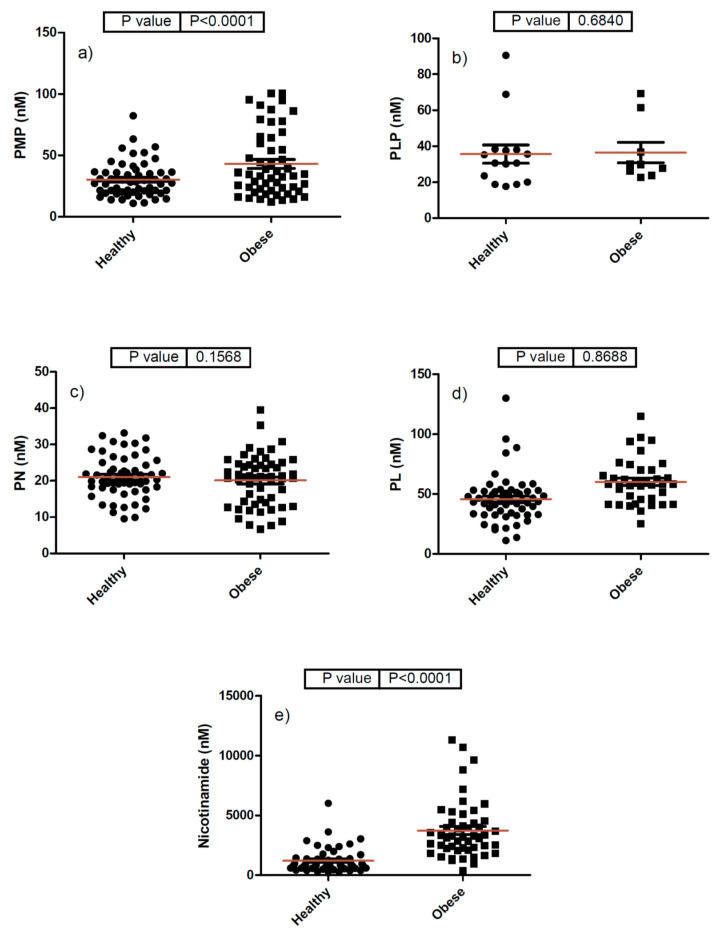
Comparative analysis of various B6 vitamers and B3 (nicotinamide) in healthy and obese Emirati populations (panels (**a**–**e**)) refer to PMP, PLP, PN, PL, and nicotinamide, respectively.

A comparative analysis was also carried out between the concentrations of the analytes found in the plasma samples of the healthy and obese patients in order to see if there were any significant differences between the two Emirati populations. As can be seen in Table 6 and Figure 2, obese Emirati patients showed significantly higher average concentrations of PMP (*p* = 0.002), PL (*p* = 0.0006), and nicotinamide (*p* < 0.0001) than healthy patients, as judged by a Student’s t-test analysis. No significant differences in the plasma concentrations of PLP and PN were observed between the healthy and obese Emirati populations. The exact implications and reasons behind these significant differences are not clear. However, it is well known that ever since the late 1930s, when the fortification of foods and supplements by synthetic vitamins started, vitamin intake has significantly increased. The vitamin paradox in obesity may reflect excess vitamin intake rather than vitamin deficiency given that there is a correlation between high vitamin intake and increased obesity [47]. These interesting differences could also be related to various factors, such as vitamin supplementation, higher caloric and food intake, other metabolic imbalances, and single nucleotide polymorphisms or genetic differences. The exact nature and cause of these findings will be carefully examined in future studies involving larger population sizes. Additionally, these data could also be correlated with gender, body weight, and other plasma parameters, such as complete blood count, HbA1c, interleukins, and liver function enzymes. Nevertheless, this initial pilot study points to the need and value of studying the plasma concentration of vitamers in metabolically distinct populations.

We also compared our data of the healthy population with two representative data sets of vitamin concentrations in healthy human plasma previously published by Redeuil et al. in 2015 [41] and Midttun et al. in 2005 [48] for US and European populations, respectively (Table 7). Compared with an average normal concentration of 4.9 nM PMP for the healthy US population [41] and below the detection limit for the European population [48], we found higher PMP concentrations in healthy and obese Emirati populations, with values of 30.2 and 53.2 nM of PMP, respectively. A similar pattern was also observed for nicotinamide, where our values in the healthy and obese plasma samples (1206.5 and 3733.5 nM, respectively) were higher than the published average value of 274.4 nM for the US samples (Table 6 and Table 7). On the other hand, the average concentrations of PLP (36 and 38 nM in the healthy and obese Emirati populations, respectively) were lower than the US population (average 92 nM). However, the European population had plasma PLP concentrations (34.4 nM) that were very similar to the UAE population. Similarly, PN, and PL were found to be lower in the plasma samples of our healthy population, as compared with those in the US population that had higher levels than the Europeans. It is interesting to note that only two of the vitamin B6 vitamers (namely PLP and PL) were detected in the plasma samples of the European healthy population, as analyzed by Midttun et al. [48]. Again, the reasons for these interesting and significant differences are most-likely multi-factorial and involve polymorphisms, dietary, and lifestyle factors. Future studies with larger cohorts of patients will need to be undertaken to establish the physiological ranges of these and other vitamers for different population groups.

## 3. Materials and Methods

### 3.1. Materials

Vitamin standards and other reagents were purchased from different suppliers as follows: pyridoxal-5′-phosphate hydrate, pyridoxal hydrochloride, pyridoxamine-5′-phosphate, nicotinamide, phosphate-buffered saline, Tris(2-carboxyethyl) phosphate hydrochloride, heptafluorobutyric acid, trichloroacetic acid, formic acid, and LC-MS-grade water were purchased from Sigma-Aldrich, St. Louis, MO, USA. Pyridoxine hydrochloride was purchased from Supelco, Sigma-Aldrich, St. Louis, MO, USA and pyridoxamine dihydrochloride was purchased from Fluka, Fisher Scientific, Waltham, MA, USA. HPLC-grade acetonitrile was purchased from Merck, Sigma-Aldrich, St. Louis, MO, USA.

### 3.2. Preparation of Standard Solutions

Individual stock solutions of B6 and B3 vitamers, as well as the internal standard, were prepared at 1000 ppm (1 µg/mL) in deionized water. These stock solutions were kept in Eppendorf tubes and stored at −80 °C to avoid degradation. Working solutions of vitamins standards were prepared daily by mixing and diluting individual stock solutions in deionized water to desired concentrations (three-fold serially diluted starting from 360 ng/mL). Preparation steps were protected from light during laboratory handling by using amber tubes to prevent vitamins from degradation.

### 3.3. Plasma Sample Extraction Method

Plasma samples from the test subjects were stored at −80 °C were thawed right before the analysis. An aliquot of 300 μL was taken into an Eppendorf tube and spiked with 10 μL of the internal standard (100 ppm), and then the mixture was vortexed for 2 min. The proteins were precipitated by adding an equal volume of 0.6 N trichloroacetic acid (TCA) to produce a final TCA concentration of 0.3 N. The samples were vortexed for 2 min and then incubated for 5 min at 50 °C. The samples were then centrifuged at 11,000 rpm for 10 min at 4 °C. The resulting supernatant was filtered using a CA (Cellulose Acetate) filter (0.22 μm), transferred into HPLC amber vials, and then placed in an autosampler where the samples were kept at 4 °C and protected from light. Normally, 8 μL of each sample extract was injected into the LC-MS/MS system.

### 3.4. Liquid Chromatography and Mass Spectrometry

The LC separation of vitamins was achieved with an Agilent 1260 HPLC system on a reversed-phase column Poroshell 120 EC-C18 (Agilent Technology, Santa Clara, CA, USA) with a particle size of 2.7 μm, an inner diameter of 3.0 mm, and a length of 100 mm. The column was maintained at 35 °C and a constant flow rate of 0.4 mL/min. Two mobile phases were used: A was LC-MS-grade water containing 0.1% formic acid, and 0.1% heptafluorobutyric acid; B, which was acetonitrile containing 0.1% formic acid. The LC method was set as follows: 3 min of 100% A, followed by a 0–100% gradient of B for 3–5 min, then 100% of B for 5–8:5 min, finally 100% A for 8:6–10 min, and finally by 100% A for 5 min as a post run. The mass spectrometry analysis was performed on an Agilent 6420 Triple Quadrupole MS system in positive electrospray ionization (ESI^+^) mode. The electrospray voltage was set at 4 kV, the ion source gas 1 (a desolvation gas consisting of nitrogen 99.9%) pressure was set at 20 psi, the ion source gas 2 (a nebulizer gas consisting of nitrogen) was set at 45 psi, and the drying gas (N_2_) flow was 8 L/min at 325 °C. Table 6 shows the precursor and product ions, along with their collision energies.

### 3.5. Study Design and Sample Collection

Study participants (57 healthy and 57 obese Emiratis) were recruited from the local Tawam Hospital in Al Ain (UAE), and signed consent forms were obtained from all the volunteers, as per the UAE University ethical approval protocol number (UAEU Ref# 09/70). The demographics and BMI (Body Mass Index) values of the volunteers were as follows: the healthy group was comprised 53 females and 4 males with an average age of 33 years (minimum age = 18; maximum age = 55), with an average BMI = 30.9 ± 0.8; the obese group was comprised of 56 females and 1 male with an average age of 35 years (minimum age = 18; maximum age = 65), with an average BMI = 33.9 ± 0.3). Plasma was prepared immediately from 10 mL of blood drawn from fasting volunteers and then stored in −80 °C. For the purpose of this preliminary study, a margin of error of 11% and a confidence level of 90% were chosen, which corresponded to an ideal sample size of 56 volunteers for each group (Raosoft sample size calculator).

## 4. Conclusions

In summary, the results presented here summarize the development of a rapid, sensitive, and robust LC-MS/MS-based assay for the simultaneous quantification of six different vitamers in human plasma. The method involves the simple, single step precipitation-based extraction of vitamins from human plasma for the subsequent analysis by an MRM-based LC-MS/MS method. This technique was subsequently used to analyze plasma samples taken from 57 healthy and 57 obese Emirati patients from a local hospital. We observed significant differences in the plasma vitamin B6 and B3 concentrations between the healthy and obese Emirati samples. Additionally, our results showed that B6 vitamers, as well as nicotinamide concentrations in the healthy Emirati population, were significantly different than those published in the literature for Western populations. The reasons behind this interesting finding will be the focus of future studies. It will also be interesting to see if the increased levels of B6 vitamers and vitamin B3 are correlated with any physiological imbalances or disease states in obese patients.

## Figures and Tables

**Table 1:** Names, structures, precursor, and product ions of all the analytes used in this study.

Name	Vitamer Structure	Mass (g/mol)	Precursor Ion [M + H]^+^ (*m*/*z*)	Product Ion [M + H]^+^ (*m*/*z*)	Fragmentor Voltage (V)	Collision Energy (eV)
Pyridoxal-5′-phosphate (PLP)	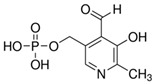 C_8_H_10_NO_6_P	247	248	149.7	45	15
Pyridoxal hydrochloride (PL)	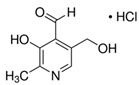 C_8_H_9_NO_3_ · HCl	203.63 167.06 (-HCl)	168	149.9	94	10
Pyridoxamine dihydrochloride (PM)	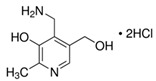 C_8_H_12_N_2_O_2_ · 2HCl	241.11 168.09 (-2HCl)	169	152	45	10
Pyridoxamine-5′-phosphate (PMP)	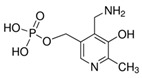 C_8_H_13_N_2_O_5_P	248	249	232.1	94	10
Pyridoxine hydrochloride (PN)	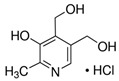 C_8_H_11_NO_3_ · HCl	205.64 169.07 (-HCl)	170	151.9	94	10
Nicotinamide	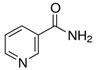 C_6_H_6_N_2_O	122.12	123	80.2	94	20
Pyridoxine-(methyl-d3) hydrochloride	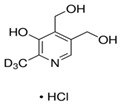 C_8_D_3_H_8_NO_3_·HCl	208.6	173	155	94	10

**Table 2:** Percentage recovery of all analytes in simulated plasma using trichloroacetic acid (TCA) and 50 °C (5 min) incubation.

Analyte	Recovery (%)	Standard Deviation
PLP	121.3	19.2
PM	82.5	1.8
PL	118.0	25.6
PN	102.8	2.6
PMP	100.8	34.1
Nicotinamide	83.6	9.2
PN-D3	95.2	2.6

**Table 3:** The lower limit of detection (LLOD) and the lower limit of quantification (LLOQ) of each analyte.

Analyte	LLOD (pg)	LLOQ (pg)
PLP	0.66	56.0
PL	6.0	18.4
PMP	6.0	18.4
PM	18.0	56.0
PN	2.0	6.0
Nicotinamide	4400	4400

**Table 4:** Quantification of B3 and B6 vitamers in 57 healthy plasma samples.

	PMP	PLP	PN	PM	PL	Nicotinamide
Sample	Concentration (nM)					
1	42.9	n.d. *	25.6	n.d.	20.3	1365.0
2	36.5	35.3	25.0	n.d.	84.3	544.9
3	21.0	n.d.	18.1	n.d.	66.8	2600.6
4	35.9	30.6	13.3	n.d.	53.8	1035.9
5	29.7	n.d.	19.2	n.d.	24.5	2492.7
6	21.4	23.5	14.9	n.d.	33.6	1338.1
7	30.7	17.6	12.7	n.d.	34.3	776.9
8	20.0	n.d.	12.3	n.d.	32.6	949.6
9	18.6	18.8	13.3	n.d.	23.8	2881.2
10	36.3	n.d.	11.3	n.d.	27.5	1138.4
11	21.6	n.d.	9.9	n.d.	31.1	3032.2
12	21.4	n.d.	15.7	n.d.	21.6	588.1
13	13.8	n.d.	17.0	n.d.	22.6	1143.8
14	17.1	38.4	9.5	n.d.	44.5	393.9
15	18.1	n.d.	22.4	n.d.	13.8	884.8
16	28.2	n.d.	23.2	n.d.	51.4	491.0
17	23.7	n.d.	18.4	n.d.	53.3	841.7
18	34.2	n.d.	22.7	n.d.	50.2	286.0
19	33.6	n.d.	16.3	n.d.	44.6	701.4
20	35.0	20.0	20.9	n.d.	58.6	1251.7
21	23.3	n.d.	30.8	n.d.	57.7	458.6
22	30.7	90.5	28.7	n.d.	130.1	n.d.
23	30.9	n.d.	21.6	n.d.	46.7	669.0
24	27.2	30.6	21.6	n.d.	53.6	1381.2
25	38.3	n.d.	19.8	n.d.	42.7	415.4
26	29.3	n.d.	19.4	n.d.	43.9	577.3
27	45.1	n.d.	19.9	n.d.	44.0	863.3
28	19.0	n.d.	21.9	n.d.	11.3	604.3
29	52.2	n.d.	18.3	n.d.	53.0	750.0
30	56.9	n.d.	13.1	n.d.	32.7	1084.5
31	82.3	38.0	17.5	n.d.	60.1	825.5
32	47.4	n.d.	21.0	n.d.	38.5	1165.4
33	51.7	n.d.	19.5	n.d.	32.2	1764.3
34	21.4	n.d.	22.0	n.d.	46.5	2390.2
35	26.6	18.8	19.4	n.d.	37.7	6005.1
36	35.9	68.9	19.1	n.d.	95.9	3620.3
37	41.2	n.d.	19.1	n.d.	32.5	830.9
38	27.4	n.d.	22.2	n.d.	42.0	965.8
39	13.8	n.d.	19.9	n.d.	42.0	1705.0
40	11.3	n.d.	22.8	n.d.	39.7	690.6
41	13.8	n.d.	20.3	n.d.	41.2	572.7
42	10.9	n.d.	21.7	n.d.	47.9	793.1
43	20.2	n.d.	21.0	n.d.	32.9	2303.8
44	26.6	n.d.	17.5	n.d.	48.9	n.d.
45	14.6	n.d.	19.3	n.d.	50.7	566.5
46	19.2	n.d.	22.1	n.d.	52.1	1348.9
47	35.9	37.6	24.3	n.d.	88.8	1980.1
48	15.9	n.d.	26.6	n.d.	43.3	n.d.
49	63.3	n.d.	27.1	n.d.	41.2	588.1
50	16.7	n.d.	28.2	n.d.	52.3	372.3
51	28.4	n.d.	26.0	n.d.	49.5	755.4
52	22.7	n.d.	30.1	n.d.	52.2	1429.8
53	21.4	n.d.	31.8	n.d.	49.0	302.1
54	55.9	35.6	32.4	n.d.	58.2	388.5
55	29.9	n.d.	33.2	n.d.	48.4	275.2
56	43.1	n.d.	28.5	n.d.	36.0	825.5
40	11.3	n.d.	22.8	n.d.	39.7	690.6
Average	30.2	36.0	21.0	n.d	45.8	1206.5
Max	82.3	90.5	33.2	n.d	130.1	6005.1
Min	10.9	n.d.	9.5	n.d	11.3	275.2

* n.d.: not detected.

**Table 5:** Quantification of B3 and B6 vitamers in 57 obese plasma samples.

	PMP	PLP	PN	PM	PL	Nicotinamide
Sample	Concentration (nM)					
1	12.0	n.d. *	24.7	n.d.	51.5	5471.0
2	34.4	n.d.	20.8	n.d.	n.d.	1823.7
3	13.6	n.d.	21.7	n.d.	48.4	1348.9
4	n.d.	n.d.	39.5	n.d.	n.d.	n.d.
5	64.3	26.2	20.9	n.d.	114.9	2314.6
6	27.8	n.d.	20.1	n.d.	58.3	1246.3
7	69.1	n.d.	21.0	n.d.	46.4	3118.6
8	14.2	n.d.	26.3	n.d.	62.2	1516.1
9	79.2	29.8	25.8	n.d.	74.7	2514.3
10	37.1	n.d.	23.8	n.d.	57.0	n.d.
11	36.1	30.2	27.2	n.d.	41.7	n.d.
12	28.7	n.d.	24.5	n.d.	45.4	n.d.
13	46.4	n.d.	23.4	n.d.	57.9	n.d.
14	15.0	n.d.	23.6	n.d.	n.d.	2044.9
15	14.4	n.d.	30.8	n.d.	n.d.	2217.5
16	24.1	n.d.	25.8	n.d.	n.d.	2854.2
17	31.3	36.8	21.2	n.d.	70.0	3177.9
18	77.9	n.d.	35.3	n.d.	63.1	2082.6
19	15.9	69.3	28.7	n.d.	95.1	3679.7
20	33.2	n.d.	24.4	n.d.	41.2	3323.6
21	20.6	n.d.	26.1	n.d.	57.6	n.d.
22	24.3	61.5	21.3	n.d.	97.3	1818.3
23	22.5	n.d.	20.3	n.d.	41.4	5956.5
24	18.6	n.d.	21.1	n.d.	41.1	6167.0
25	59.8	n.d.	28.0	n.d.	40.7	2271.5
26	42.9	n.d.	29.1	n.d.	86.2	1645.6
27	18.3	n.d.	22.4	n.d.	41.5	4526.8
28	33.2	n.d.	25.0	n.d.	40.0	3275.0
29	21.2	n.d.	23.4	n.d.	70.1	4402.7
30	16.1	n.d.	20.6	n.d.	54.3	3258.8
31	77.3	n.d.	12.6	n.d.	56.7	5287.5
32	32.4	22.7	11.8	n.d.	62.6	8810.7
33	94.8	n.d.	14.3	n.d.	59.0	2401.0
34	86.0	n.d.	14.8	n.d.	35.8	1494.5
35	38.8	n.d.	24.3	n.d.	93.9	5406.2
36	243.9	n.d.	18.0	n.d.	63.6	9625.4
37	138.3	n.d.	20.7	n.d.	65.3	11,303.4
38	44.3	n.d.	12.1	n.d.	76.3	5109.5
39	100.6	n.d.	12.0	n.d.	58.3	4337.9
40	95.4	n.d.	6.7	n.d.	75.6	3345.2
41	101.0	n.d.	n.d.	n.d.	58.3	4105.9
42	47.8	n.d.	20.7	n.d.	n.d.	n.d.
43	25.6	n.d.	17.6	n.d.	n.d.	3998.0
44	34.8	n.d.	21.8	n.d.	n.d.	3064.6
45	90.9	n.d.	16.4	n.d.	n.d.	3933.3
46	168.2	27.8	21.3	n.d.	60.6	1386.6
47	17.5	n.d.	19.2	n.d.	n.d.	7181.3
48	27.2	n.d.	12.7	n.d.	25.2	10,693.7
49	191.9	n.d.	7.7	n.d.	n.d.	372.3
50	65.3	n.d.	13.5	n.d.	n.d.	2832.6
51	53.8	n.d.	15.4	n.d.	n.d.	2498.1
52	19.8	n.d.	14.0	n.d.	n.d.	3884.7
53	39.0	n.d.	7.8	n.d.	n.d.	3965.6
54	87.2	n.d.	12.9	n.d.	n.d.	933.4
55	26.8	n.d.	9.5	n.d.	n.d.	2476.5
56	54.6	n.d.	11.4	n.d.	n.d.	2633.0
57	23.7	n.d.	8.8	n.d.	n.d.	3539.4
Average	53.2	38.0	20.1	n.d.	60.2	3733.5
Max	243.9	69.3	39.5	n.d.	114.9	11,303.4
Min	12.0	n.d.	6.7	n.d.	n.d	372.3

* n.d.: not detected.

**Table 6:** Concentration of various B6 vitamers and nicotinamide in healthy and obese Emirati populations.

Analyte (nM)	Healthy Plasma			Obese Plasma		
	Average	Max	Min	Average	Max	Min
PMP	30.2	82.3	10.9	53.2	243.9	12.0
PLP	36.0	90.5	n.d. *	38.0	69.3	n.d.
PN	21.0	33.2	9.5	20.1	39.5	6.7
PM	n.d.					
PL	45.8	130.1	11.3	60.2	114.9	n.d.
Nicotinamide	1206.5	6005.1	275.2	3733.5	11303.4	372.3

* n.d.: not detected.

**Table 7:** Comparison between the concentrations of all analytes in the plasma samples of a healthy population and those mentioned in the literature.

Analyte (nM)	Healthy Emirati Plasma			US Population [41]			European Population [48]		
	Average	Max	Min	Average	Max	Min	Average	Max	Min
PMP	30.2	82.3	10.9	4.9	7.6	2.1	Not detected		
PLP	36.0	90.5	n.d.	92.1	163.3	20.9	34.4	102.3	17.0
PN	21.0	33.2	9.5	142.8	285.4	0.2	Not detected		
PM	Not detected			4.1	7.7	0.4	Not detected		
PL	45.8	130.1	11.3	118.4	233.5	3.2	9.9	28.2	5.7
Nicotinamide	1206.5	6005.1	275.2	274.4	479.6	69.1	Not included		

## Supplementary Materials

The following are available online. Figure S1: Concentrations of PMP, PL, PN, PL and Nicotinamide in healthy Emirati population (*n* = 57), Figure S2: Concentrations of PMP, PL, PN, PL and Nicotinamide in obese Emirati population (*n* = 57), Table S1: Effect of incubation temperature on TCA-precipitated release of vitamers from spiked simulated plasma, Table S2: Intra-day and inter-day accuracy, precision and linear range of the partially validated LC-MSMS method.

## Abbreviations

PL	Pyridoxal
PLP	Pyridoxal 5′-phosphate
PM	Pyridoxamine
PMP	Pyridoxamine 5′-phosphate
PN	Pyridoxine; PN-d3: Pyridoxine - (methyl-d3)
TCA	Trichloroacetic acid
n.d.	Not detected
LC-MS/MS	Liquid Chromatography tandem Mass Spectrometry
CVD	Cardiovascular disease
BMI	Body Mass Index
