# Supplementary material for: Significantly Elevated Levels of Plasma Nicotinamide, Pyridoxal, and Pyridoxamine Phosphate Levels in Obese Emirati Population: A Cross-Sectional Study
Source: Molecules. 2020 Aug 28;25(17):3932. doi: 10.3390/molecules25173932 (PMC7504312; doi:10.3390/molecules25173932)
Supplement: Supplementary file 1 [file molecules-25-03932-s001.pdf]

molecules MDPI

Supplementary file 1

Significantly elevated levels of plasma Nicotinamide, Pyridoxal and Pyridoxamine Phosphate levels in obese Emirati population: a cross-sectional study

Ghada Rashad Ibrahim[1], Iltaf Shah[1], Salah Gariballa[2], Javed Yasin[2], James Barker[3] and Syed Salman Ashraf*[4]

[1] Department of Chemistry, College of Science, UAE University, Al Ain, UAE.
[2] Department of Internal Medicine, College of Medicine, UAE University, Al Ain, UAE
[3] Department of Chemical and Pharmaceutical Sciences, School of Life Sciences, Pharmacy and Chemistry, Kingston University
[4] Department of Chemistry, College of Arts and Sciences, Khalifa University, Abu Dhabi, UAE.
* Correspondence: Dr. S. Salman Ashraf, Department of Chemistry, College of Arts and Sciences, Khalifa University, P O Box 127788, Abu Dhabi, UAE. Tel: +971 2 501 8483

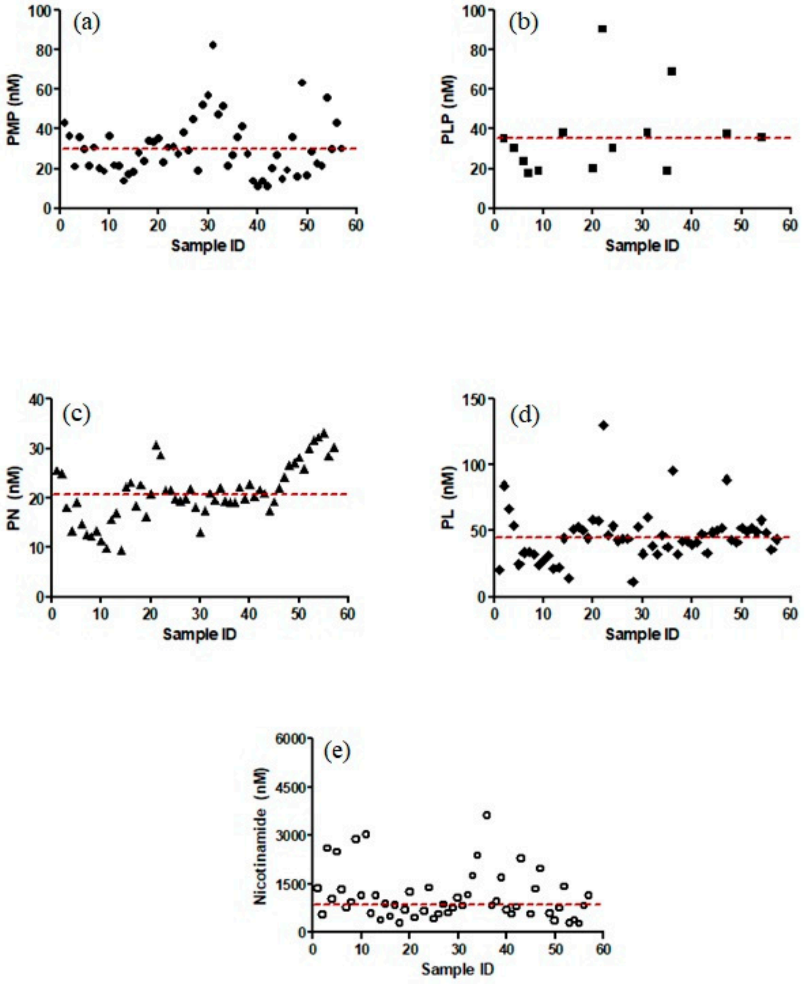

Figure S1. Concentrations of PMP, PL, PN, PL and Nicotinamide in healthy Emirati population (n=57).

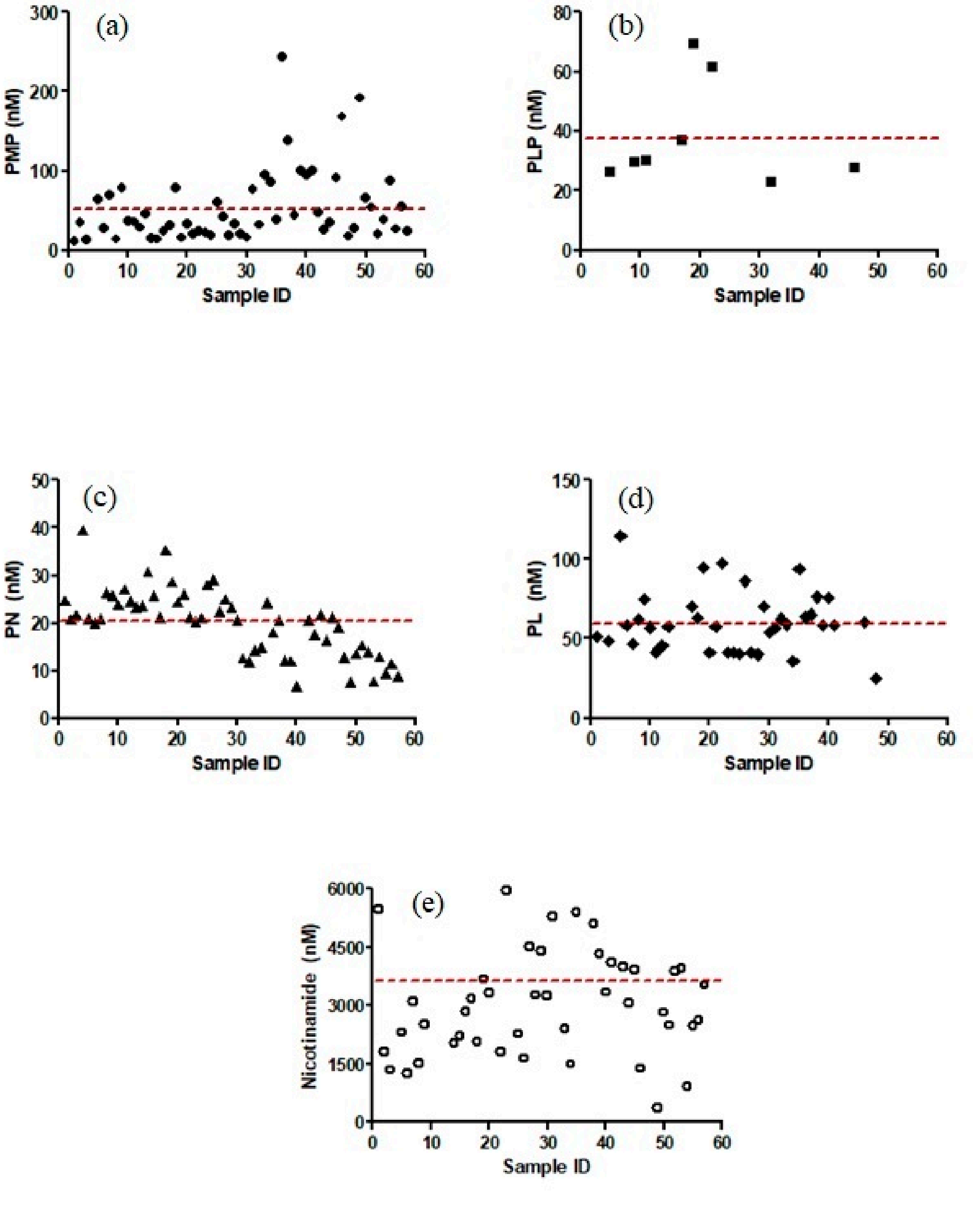

Figure S2. Concentrations of PMP, PL, PN, PL and Nicotinamide in obese Emirati population (n=57).

Table S1: Effect of incubation temperature on TCA-precipitated release of vitamers from spiked simulated plasma

Analyte	0^0C (1 hour)	25^0C (1 hour)	50^0C (5 min)
	Area Under the Curve (AUC)		
**PMP**	16,599	20,120	21,931
**PLP**	36,480	41,076	41,229
**PN**	222,146	234,575	245,534
**PM**	212,681	243,573	264,702
**PL**	230,989	277,679	275,796
**Nicotinamide**	240,934	313,138	352,393

Table S2: Intra-day and inter-day accuracy, precision and linear range of the partially validated LC-MSMS method

S/N	Analytes	Intra-Day		Inter-day		Linear Range	r2
		Precision (%CV)	% Accuracy	Precision (%CV)	% Accuracy	(ng/mL)	
1	PLP	12.1	115.2	6.0	103.3	0.056-360	0.970
2	PL	13.4	104.7	5.2	112.7	0.0184-360	0.974
3	PMP	11.4	129.3	8.4	86.1	0.0184-360	0.978
4	PM	3.0	100.3	9.8	92.5	0.056-360	0.998
5	PN	2.3	99.8	43.5	101.2	0.006-111	0.985
6	Nicotinamide	7.5	102.3	7.3	93.9	4.4-360	0.940
